# Teaching Adequate Prehospital Use of Personal Protective Equipment During the COVID-19 Pandemic: Development of a Gamified e-Learning Module

**DOI:** 10.2196/20173

**Published:** 2020-06-12

**Authors:** Mélanie Suppan, Birgit Gartner, Eric Golay, Loric Stuby, Marion White, Philippe Cottet, Mohamed Abbas, Anne Iten, Stephan Harbarth, Laurent Suppan

**Affiliations:** 1 Division of Anesthesiology Department of Anesthesiology, Clinical Pharmacology, Intensive Care and Emergency Medicine University of Geneva Hospitals and Faculty of Medicine Geneva Switzerland; 2 Division of Emergency Medicine Department of Anesthesiology, Clinical Pharmacology, Intensive Care and Emergency Medicine University of Geneva Hospitals and Faculty of Medicine Geneva Switzerland; 3 Genève TEAM Ambulances Geneva Switzerland; 4 Infection Control Program and WHO Collaborating Centre on Patient Safety University of Geneva Hospitals and Faculty of Medicine Geneva Switzerland

**Keywords:** personal protective equipment, electronic learning, gamification, prehospital, COVID-19

## Abstract

**Background:**

The coronavirus disease (COVID-19) pandemic has led to increased use of personal protective equipment (PPE). Adequate use of this equipment is more critical than ever because the risk of shortages must be balanced against the need to effectively protect health care workers, including prehospital personnel. Specific training is therefore necessary; however, the need for social distancing has markedly disrupted the delivery of continuing education courses. Electronic learning (e-learning) may provide significant advantages because it requires neither the physical presence of learners nor the repetitive use of equipment for demonstration.

**Objective:**

Inclusion of game mechanics, or “gamification,” has been shown to increase knowledge and skill acquisition. The objective of this research was to develop a gamified e-learning module to interactively deliver concepts and information regarding the correct choice and handling of PPE.

**Methods:**

The SERES framework was used to define and describe the development process, including scientific and design foundations. After we defined the target audience and learning objectives by interviewing the stakeholders, we searched the scientific literature to establish relevant theoretical bases. The learning contents were validated by infection control and prehospital experts. Learning mechanics were then determined according to the learning objectives, and the content that could benefit from the inclusion of game mechanics was identified.

**Results:**

The literature search resulted in the selection and inclusion of 12 articles. In addition to gamification, pretesting, feedback, avoiding content skipping, and demonstrations using embedded videos were used as learning mechanics. Gamification was used to enhance the interactivity of the PPE donning and doffing sequences, which presented the greatest learning challenges. The module was developed with Articulate Storyline 3 to ensure that it would be compatible with a wide array of devices, as this software generates HTML5-compatible output that can be accessed on smartphones, tablets, and regular computers as long as a recent browser is available.

**Conclusions:**

A gamified e-learning module designed to promote better knowledge and understanding of PPE use among prehospital health care workers was created by following the SERES framework. The impact of this module should now be assessed by means of a randomized controlled trial.

## Introduction

### Background and Importance

Acquisition and regular updating of specific knowledge and skills are paramount in the context of an evolving major health crisis. However, the need for social distancing due to the coronavirus disease (COVID-19) pandemic has markedly disrupted continuing medical education [1]. Adequate supply and use of personal protective equipment (PPE) is more critical than ever because the risk of shortages must be balanced against the need to efficiently protect health care workers, including prehospital personnel [2,3]. Indeed, contamination of these professionals may lead to further dissemination of the disease, including among frail patients being transported in the closed cell space of an ambulance, and it can affect human resources if paramedics and prehospital emergency physicians are also infected [4-6]. This in turn may decrease the ability of emergency medical services (EMS) to fulfill their mission.

In this challenging context, electronic learning (e-learning) may provide significant advantages because it requires neither the physical presence of learners nor the repetitive use of equipment for demonstration, as can be the case during live simulations [7,8]. The term e-learning is generic and refers to a host of different methods and materials [9-11]. Acquisition of knowledge and skills increases with interactivity, and this increase is even greater with the inclusion of game mechanics, or “gamification” [12,13].

### Objective

A gamified e-learning module may enhance the knowledge and skills of prehospital personnel regarding the correct choice and handling of PPE. The objective of this research was to develop an evidence-based, gamified e-learning module addressing these aspects.

## Methods

### General Design

The SERES framework was used to define and describe the development process of this gamified e-learning module, including scientific and design foundations [14].

### Scientific Foundations

#### Target Audience

To identify all the categories of health care professionals who may be expected to don PPE in the prehospital setting, the module developers interviewed chief ambulance officers, chief medical officers, paramedics, and emergency physicians working in Swiss EMS.

#### Learning Objectives

Given the general objective and the target audience, we performed individual interviews to assess specific learning objectives that the gamified e-learning module needed to fulfill. At this stage, specialists from the Geneva University Hospitals infection control program were included in the discussion.

#### Theoretical Basis

Use of game mechanics should not be a goal per se but a means to ensure that the intended learning objectives are met. We therefore searched the scientific literature using the PubMed engine with combinations (using the Boolean operator “AND”) of the medical subject headings (MeSH) keywords *serious games*, *prehospital*, and *infection control* and the non-MeSH keywords *gamification*, *e-learning*, and *electronic learning*. Potentially relevant articles were retrieved based on their titles and abstracts. References from the most authoritative and relevant articles were manually screened to identify papers our initial search may have overlooked. Articles that were not written in English or French were excluded.

Factors contributing to learner engagement as well as to skill and knowledge acquisition were collected before being individually assessed according to their potential impact. As game mechanics may not apply to all aspects of knowledge acquisition, it was necessary to identify specific elements that could benefit from the use of such mechanics. Specialists from the infection control program as well as chief ambulance and medical officers were again consulted at this stage.

#### Content Validation

Infection control specialists were included early in the design phase of the module to validate the learning content and its coherence with local COVID-19 control guidelines. Due to the rapid and incessant growth of knowledge regarding COVID-19 and the need to preserve PPE, the advice of these specialists was essential.

### Design Foundations

#### General Design

The gamified e-learning module was designed according to the scientific foundations established in the previous stage.

#### Learning Mechanics and Game Mechanics

To apply game mechanics to the learning objectives that could most benefit from this method, the learning mechanics-game mechanics (LM-GM) model proposed by Arnab [15] in 2015 was used. In this model, learning mechanics are determined according to learning objectives and are then transformed into game mechanics to achieve the intended goal. The scientific foundations for the module were therefore first translated into learning mechanics and then, when appropriate, into game mechanics.

#### Design Requirements

When making design decisions, it was necessary to consider the particular context of the COVID-19 pandemic along with the specific target population. The time taken to complete the whole module and the type of media included in the module were assessed in this regard. Ease of access to the module, including the platforms and support that could be used, was also considered.

#### Module Development

This stage required the integration of all the data collected during the scientific foundation and design foundation stages. The learning objectives were reviewed, and learning mechanics were decided according to theoretical basis. Decisions regarding gamification of specific sections of the e-learning module were reassessed. An iterative approach to the construction of the module was used, incorporating regular feedback from the different previously identified stakeholders (infection prevention and control consultants, chief ambulance officers, chief medical officers, paramedics, and emergency physicians).

#### Tool Evaluation

During the iterative development loops and at the end of the development process, the module was tested for usability according to theoretical bases acquired during the scientific foundations stage.

## Results

### Scientific Foundations

#### Target Audience

Five categories of health care professionals working in the prehospital setting were identified: emergency physicians, paramedics, emergency medical technicians, nurses, and ambulance drivers [16,17]. Although they are not strictly health care professionals, ambulance drivers are trained in providing basic life support measures and may encounter situations that require them to don PPE. Therefore, we considered a vast array of professionals who present important variability regarding both medical knowledge and technical skills.

#### Learning Objectives

The frequent guideline updates consecutive to the general increase in knowledge and understanding regarding severe acute respiratory coronavirus 2 (SARS-CoV-2), the virus that causes COVID-19, and its mode of transmission prompted the definition of specific objectives (Table 1).

#### Theoretical Basis

A literature search resulted in the selection and inclusion of 12 articles [8,9,12-15,18-22]. Based on these results, the module developers, along with the stakeholders, decided which learning mechanics would be used to fulfill the different learning objectives (Table 1).

**Figure 1:**
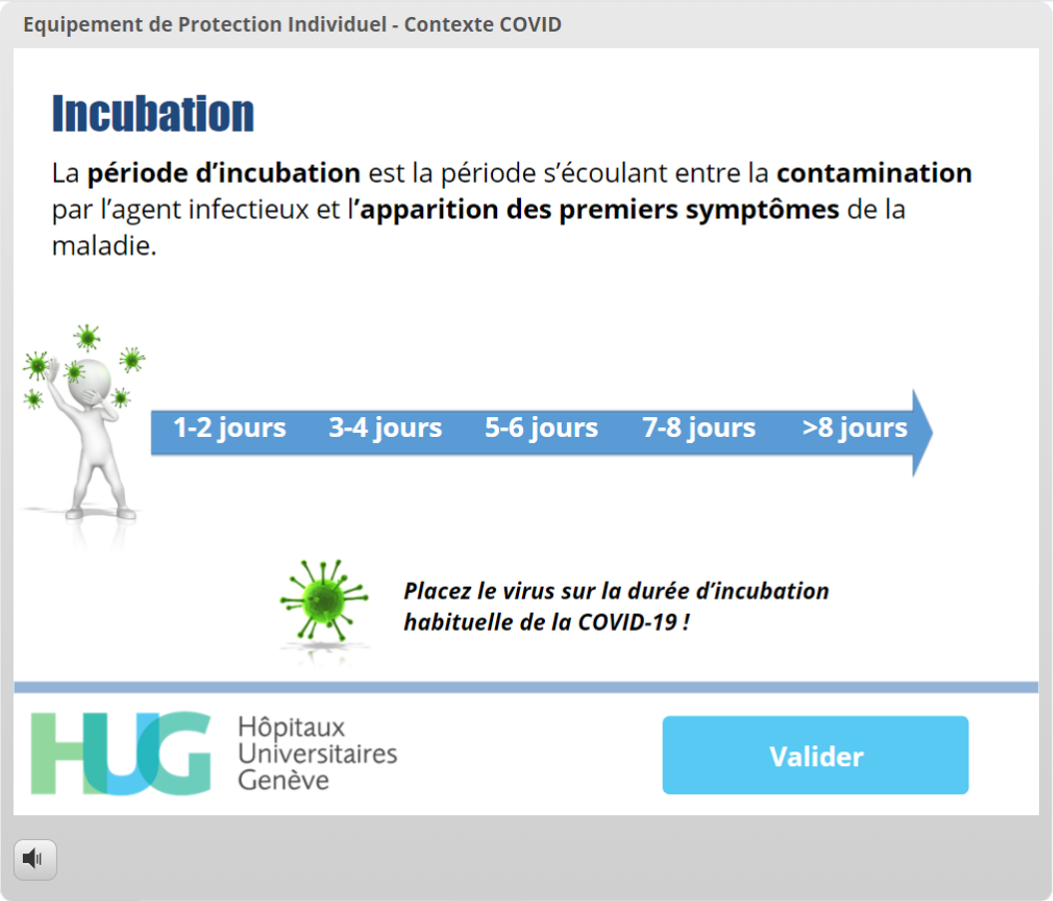
Use of pretesting to promote acquisition of knowledge regarding incubation time. The virus must be placed on the timeline before the *Valider* (Validate My Answer) button can be successfully used.

**Figure 2:**
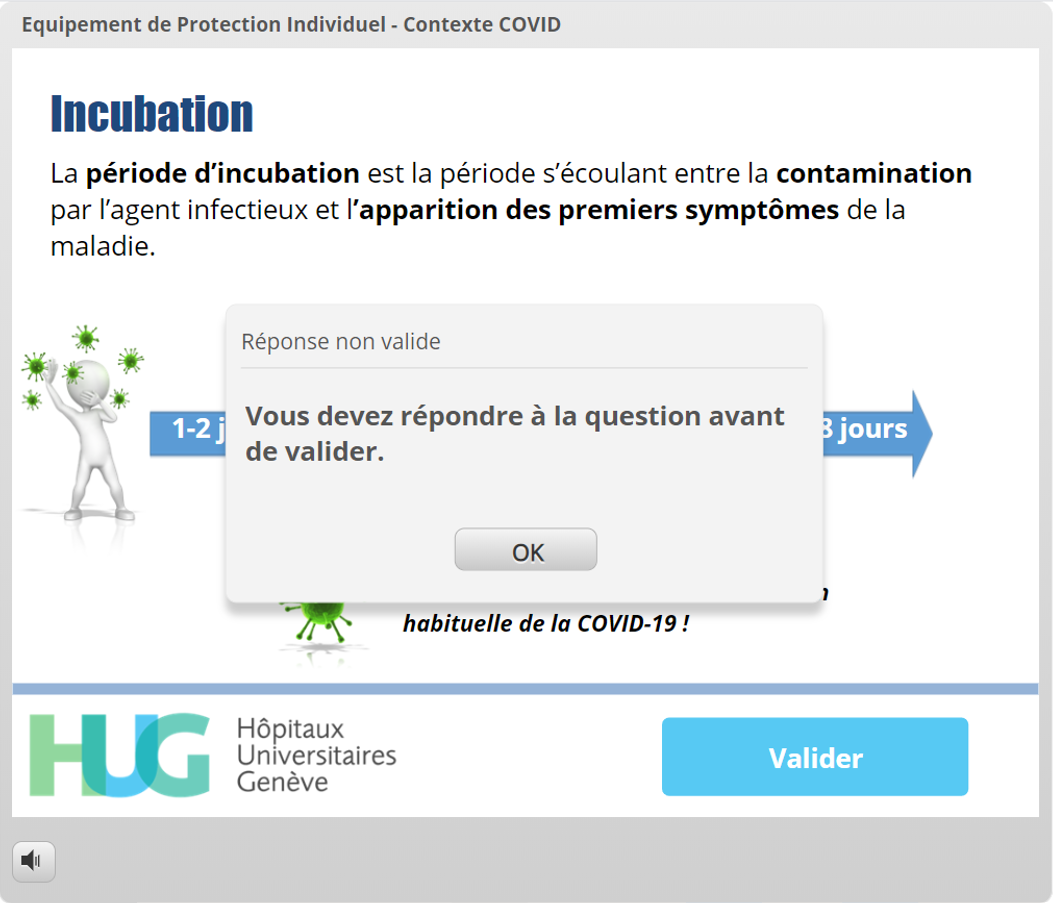
Mechanism preventing users from moving forward without completing the interaction. The learner clicked the *Valider* (Validate My Answer) button but could not proceed further because the virus had not been placed on the timeline.

**Figure 3:**
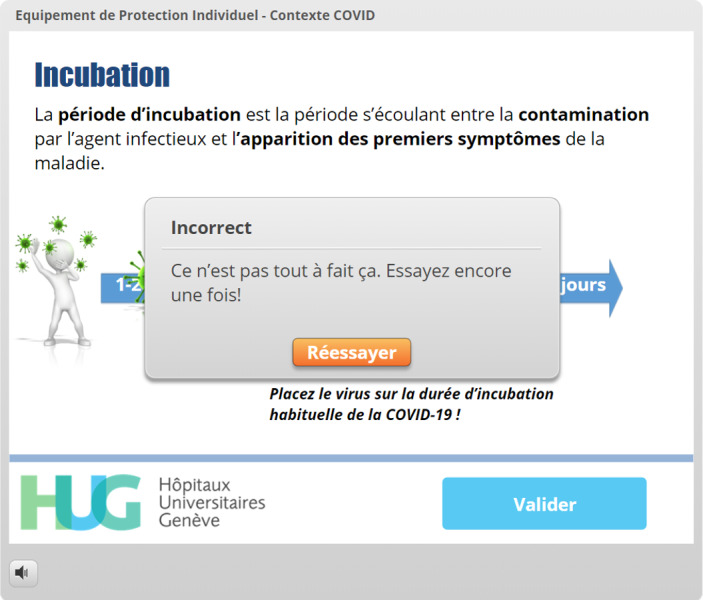
The learner has provided the wrong answer and is given the opportunity to retry. In this interaction, learners are allowed one more attempt if their first attempt fails.

**Figure 4:**
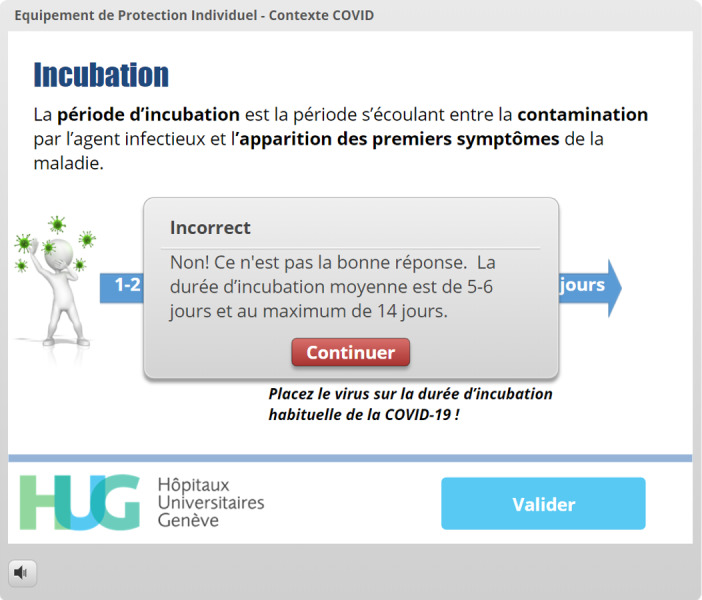
Response when the second answer attempt is also unsuccessful. Immediate feedback is provided, showing the right answer and other relevant information. The learner can now click on *Continuer* (Continue) to move forward.

A pretesting strategy was used for one of the learning objectives (incubation time), as this method has been shown to improve knowledge retention [19] (Figures 1-4). Following this type of interaction, providing the correct answer seems to be insufficient, and more detailed feedback has been shown to yield better results regarding knowledge acquisition [18].

**Figure 5:**
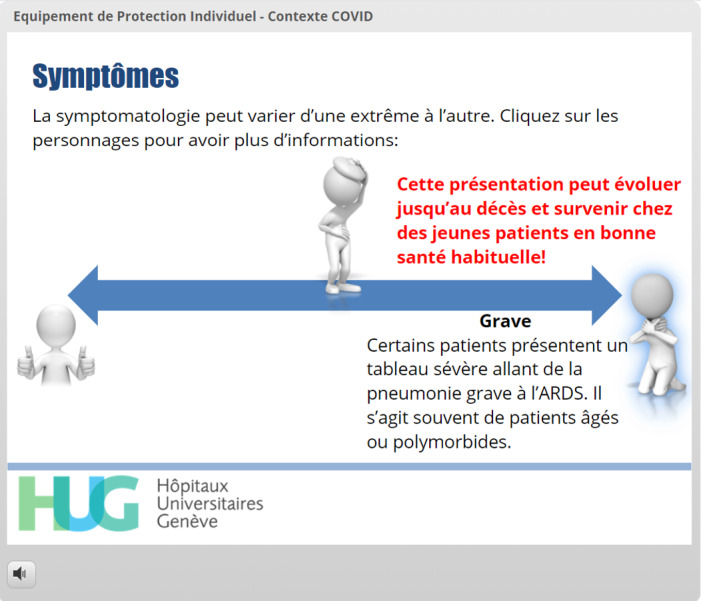
Use of triggers to avoid content skipping. The “very ill” patient on the rightmost side of the slide has been clicked and is therefore highlighted. The learning content related to this presentation is displayed; however, the *Continuer* (Continue) button does not appear because the learner must click at least one of the other patients.

**Figure 6:**
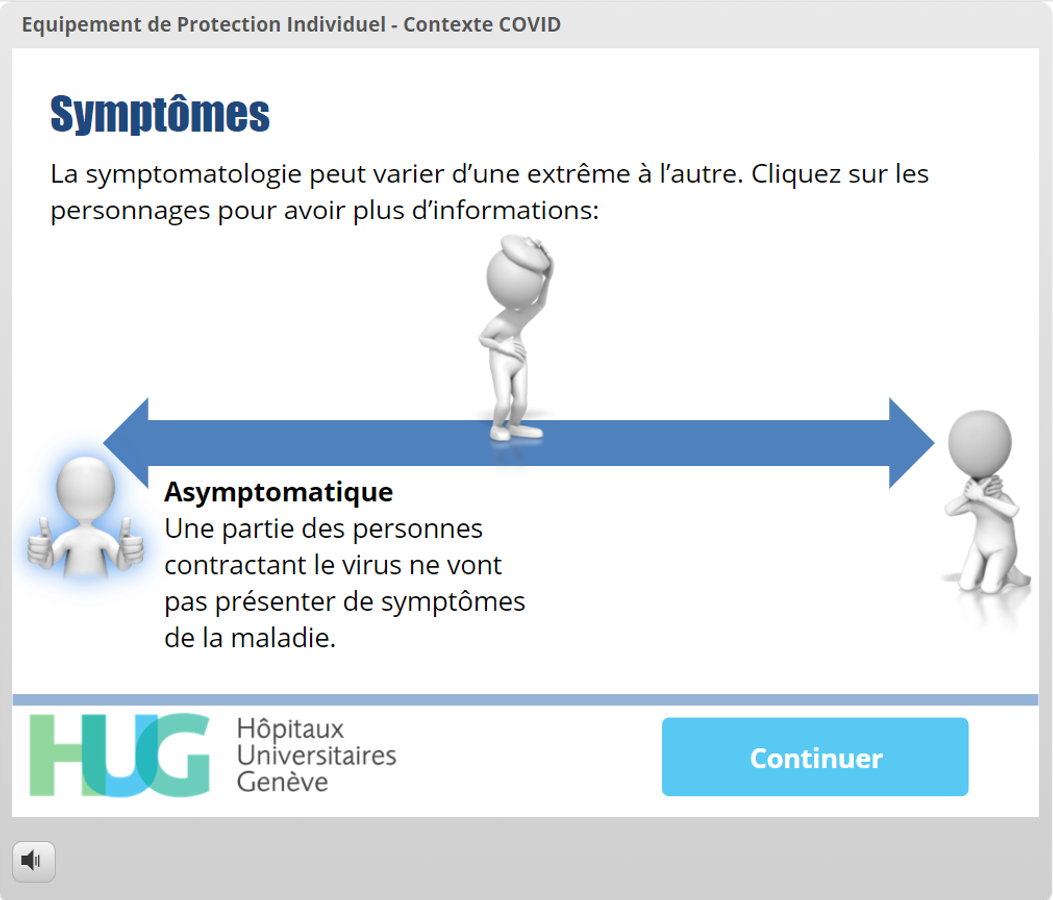
The learner has now clicked on all three patients, and the *Continuer* (Continue) button is therefore displayed, allowing the learner to move forward.

One important aspect was to prevent learners from skipping content, which would cause them to miss parts of the learning material [21] (Figures 5 and 6). In addition to setting triggers designed to restrict further access without engaging with the interactive content, the content was segmented in multiple slides and slide layers to keep the learner engaged [20].

**Figure 7:**
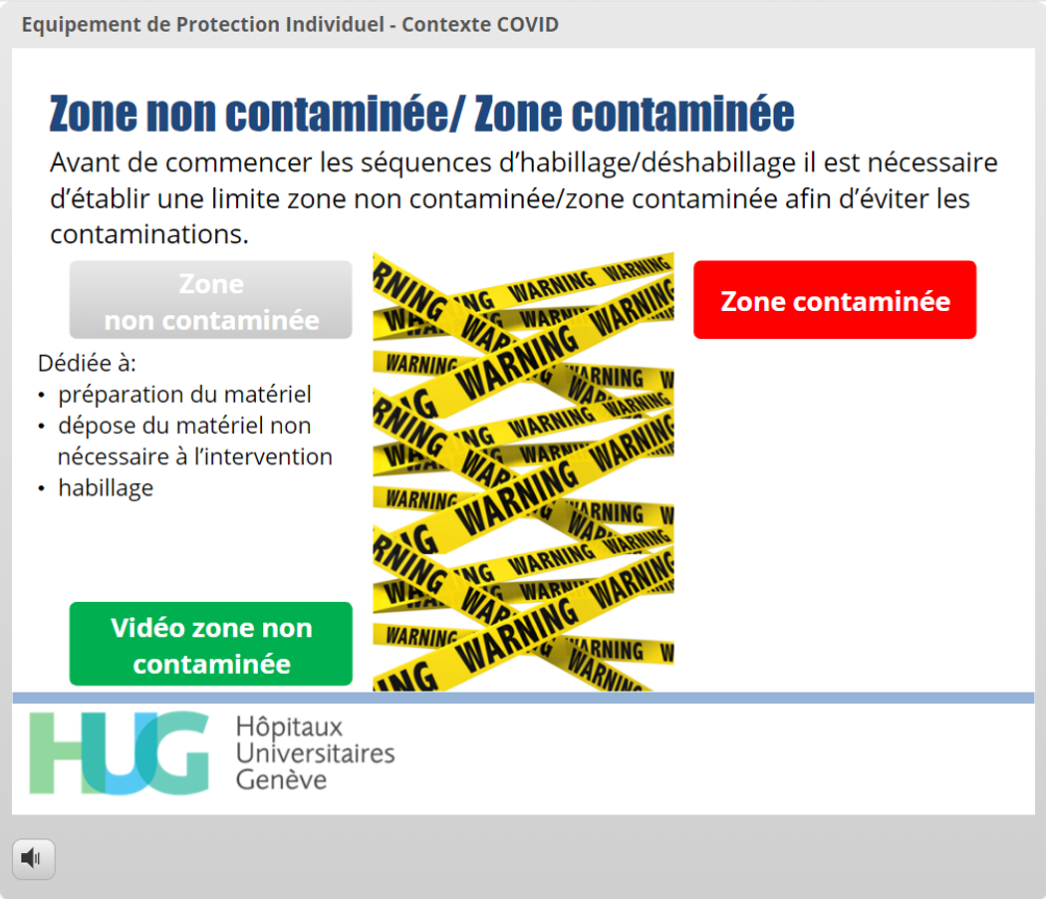
The learner has clicked on the button *Zone non contaminée* (Uncontaminated Zone). A new button, *Vidéo zone non contaminée* (Uncontaminated Zone Video), has now appeared. The *Continuer* (Continue) button is not displayed because the learner has not yet viewed all the learning material in this sequence.

**Figure 8:**
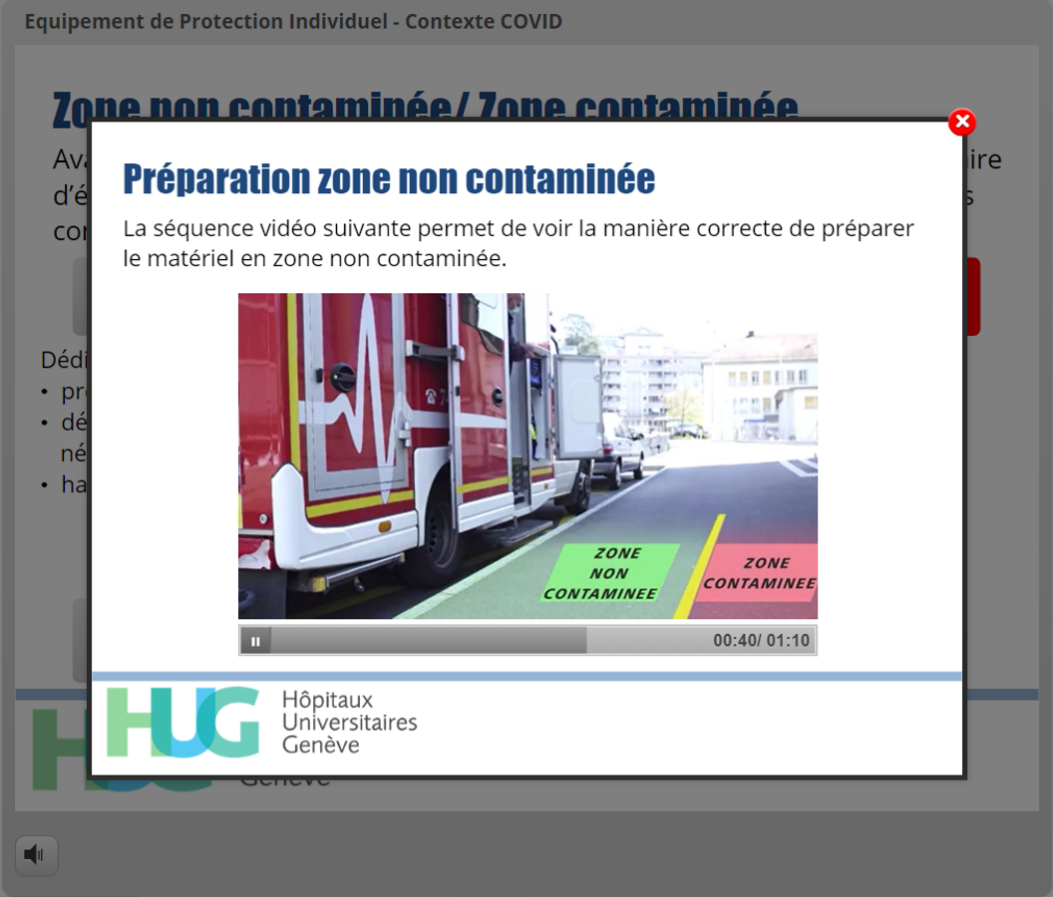
Use of embedded videos to display virtual zones. The learner has clicked on the *Vidéo zone non contaminée* (Uncontaminated Zone Video) button, and the slide containing the video is lightboxed.

As some skills are better demonstrated and some related knowledge is more readily acquired through video [9], a movie demonstrating the correct PPE donning and doffing sequences was recorded. Portions of this video were then embedded in the module, particularly in its gamified portion (Figures 7 and 8) [23].

**Table 1:** Learning objectives, learning mechanics with references, and related implementation examples.

Learning objective	Learning mechanic	Implementation
Incubation time	Pretesting and feedback [18,19]	Figures 1-4: The virus must be placed in a spot on the timeline before the “Validate My Answer” button can be successfully clicked.
Knowledge of specific definitions, virus transmission, and disease symptoms	Avoiding content skipping [20,21]	Figures 5 and 6: All three images displaying patients (healthy, somewhat ill, and very ill) must be clicked before the learner is allowed to move forward. A different layer of information is displayed depending on the image clicked.
Definition of virtual zones	Avoiding content skipping, embedded videos [9,20]	Figures 7 and 8: Buttons linking to information regarding the contaminated and noncontaminated zones are displayed. A new button is displayed with the explanation for each zone. When the button is clicked, a video sequence describing each zone is shown.
PPE items, with donning and doffing sequences	Gamification, embedded videos [9,13]	Figures 9-11: Screenshots of the gamified PPE donning sequence (see the Gamified Sequences section).

Finally, as health care personnel spend increasing amounts of time on their smartphones or tablets, and as these devices use many different and sometimes incompatible operating systems, the module was developed to be accessible on as many platforms as possible [22,24].

#### Module Development

A movie including the complete PPE donning and doffing sequences was recorded according to the latest prevention guidelines. To obtain realistic media to create the gamified donning and doffing sequences, a photo shoot was performed in front of a green screen, and the background was removed using chroma key technology [25]. Graphics such as stick figures were obtained from PresenterMedia (Eclipse Digital Imaging Inc).

The module was developed using Storyline 3 (Articulate Global), which enables publication in the HTML5 markup language; therefore, the module is compatible with most devices, including tablets and smartphones.

#### Gamified Sequences

Based on our prior decisions, game mechanics were applied to the donning and doffing sequences, which presented the greatest learning challenges. According to Bloom’s revised taxonomy, three thinking skills matched this learning objective: applying, understanding, and retention [26]. The applying skill was linked to the action/task learning mechanic, which was translated into the “selecting/collecting” and “movement” game mechanics. Understanding was promoted by questions and answers and by instantaneous feedback, which are part of both learning mechanics and game mechanics. Retention was included using the “discover,” “explore,” and “repetition” learning mechanics and the “cutscenes/story” game mechanic.

**Figure 9:**
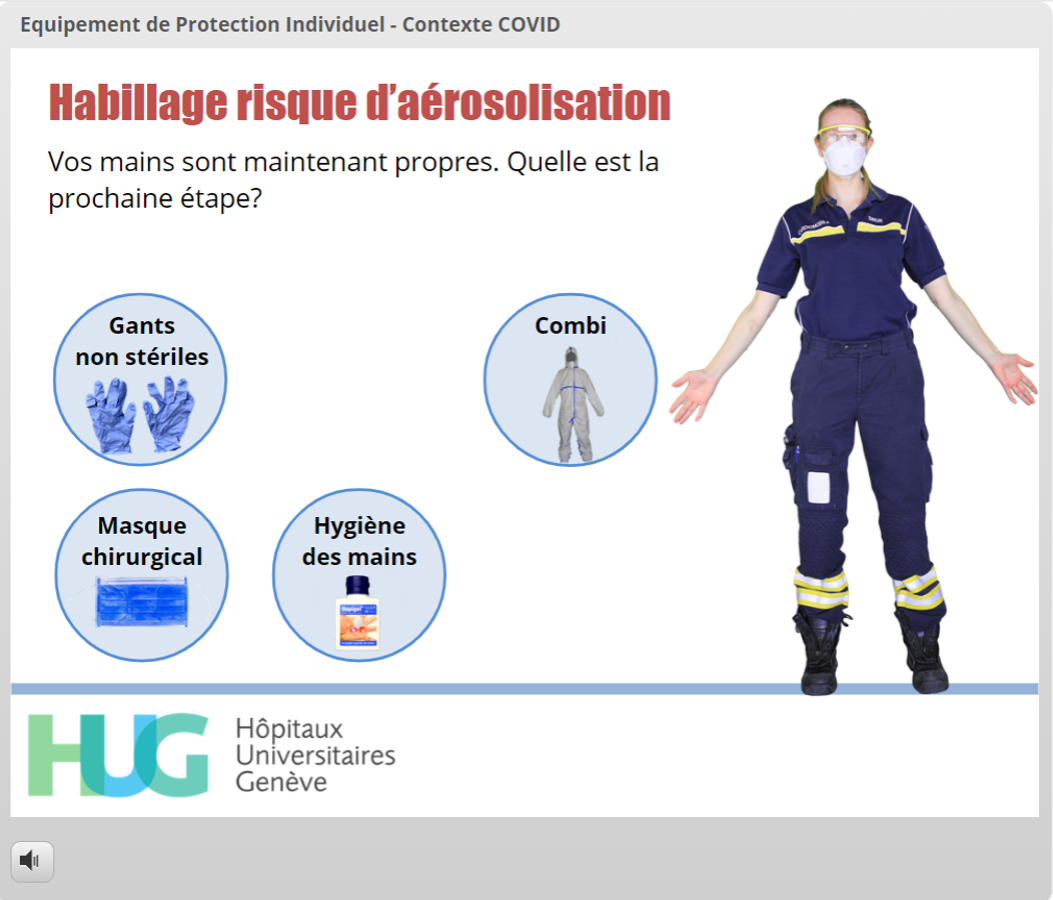
Use of gamification to facilitate acquisition of the correct sequence of donning PPE before high-risk procedures such as endotracheal intubation. The user has already correctly used the FFP2 respiratory mask and protective goggles and has proceeded with hand hygiene. The feedback is displayed at the top of the screen (*“Vos mains sont maintenant propres. Quelle est la prochaine étape?” which means “Your hands are now clean. What should the next step be?”*).

**Figure 10:**
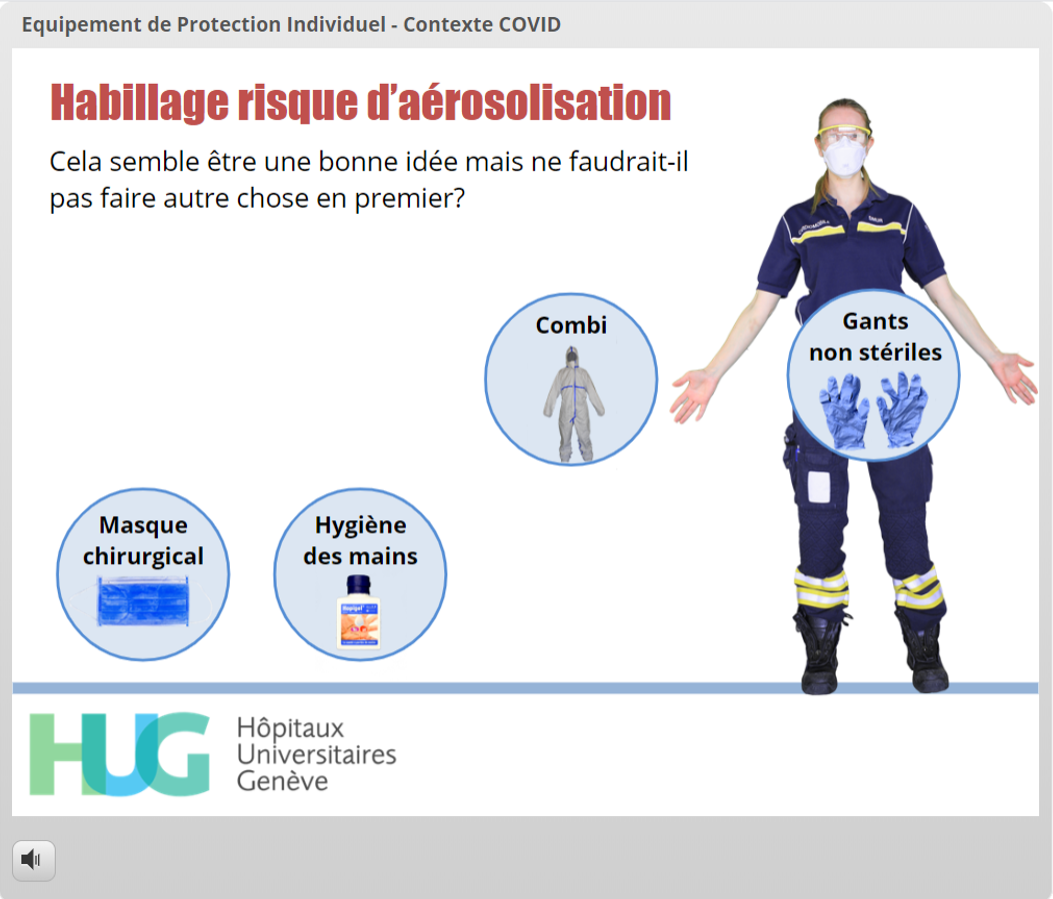
The learner has elected to try to put on gloves at this stage but is advised to try something else first without being given negative feedback (*“Cela semble être une bonne idée mais ne faudrait-il pas faire autre chose en premier?” which means “This looks like a good idea, but shouldn’t you do something else first?”*).

**Figure 11:**
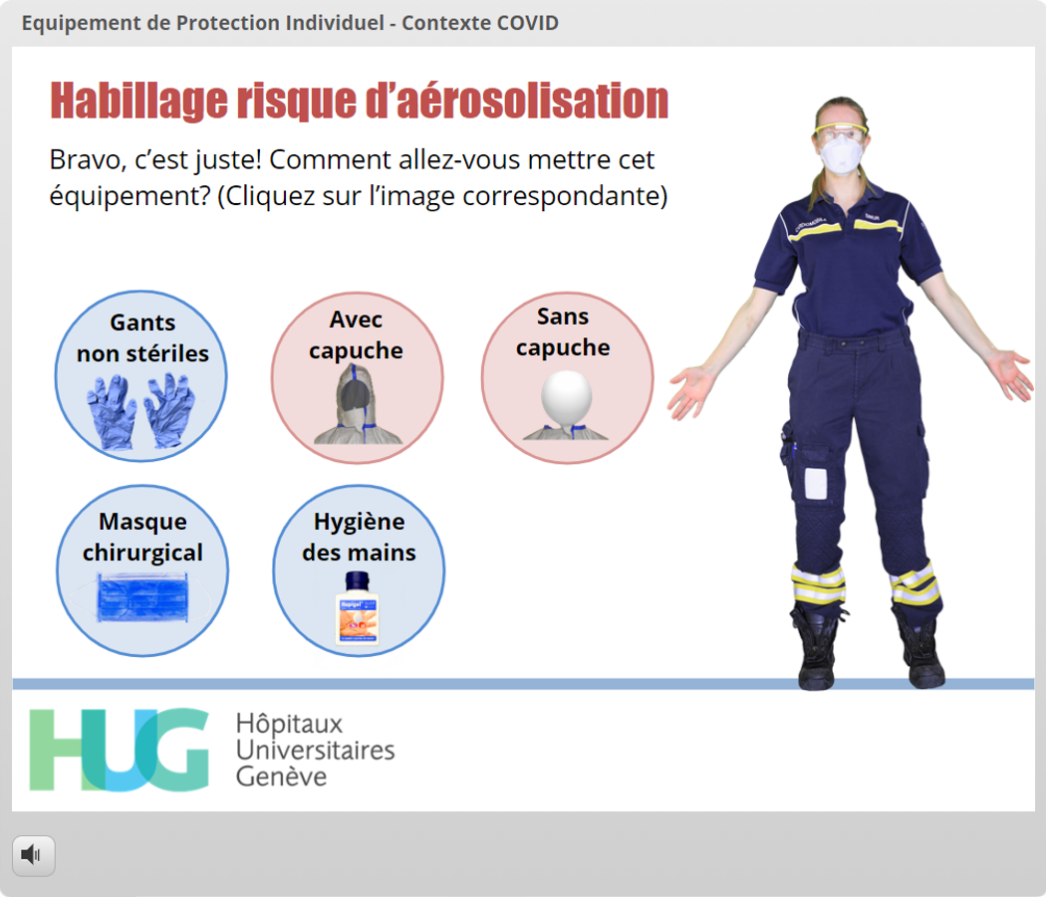
The learner has now decided to put on the overalls, which is the right answer at this stage. The positive feedback is displayed at the top of the screen (*“Bravo, c’est juste” which means “Congratulations, you’re right!”*), and two options (with or without hood) have appeared.

Highly interactive sequences using photographs and embedded videos were created for three different COVID-19 risk settings: no suspicion of COVID-19, suspected or confirmed COVID-19 with no need to perform a high-risk procedure, and need to perform a high-risk procedure regardless of COVID-19 status. For each donning sequence, the learner was given the same six choices, including five different PPE options and a hand hygiene procedure option. Each option was represented by a photograph of the material that would be used and the name or abbreviation given to this material, all of which were grouped within circles (Figures 9-11). Learners made their choices by dragging one of the options onto the photograph of the EMS provider. Immediate written feedback was provided at the top of the screen (Figures 9-11).

**Figure 12:**
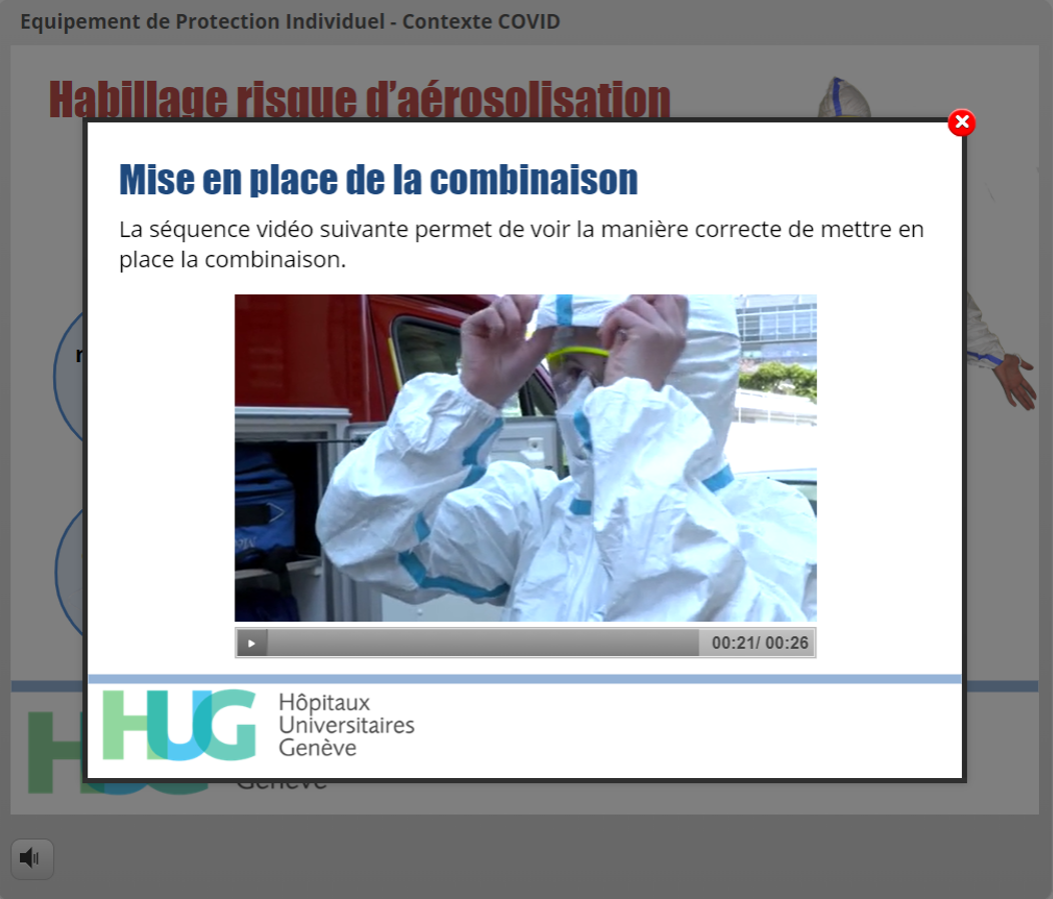
Automatic launch of a video in a lightbox upon selection of the correct PPE option. The video illustrates complex donning or doffing sequences.

**Figure 13:**
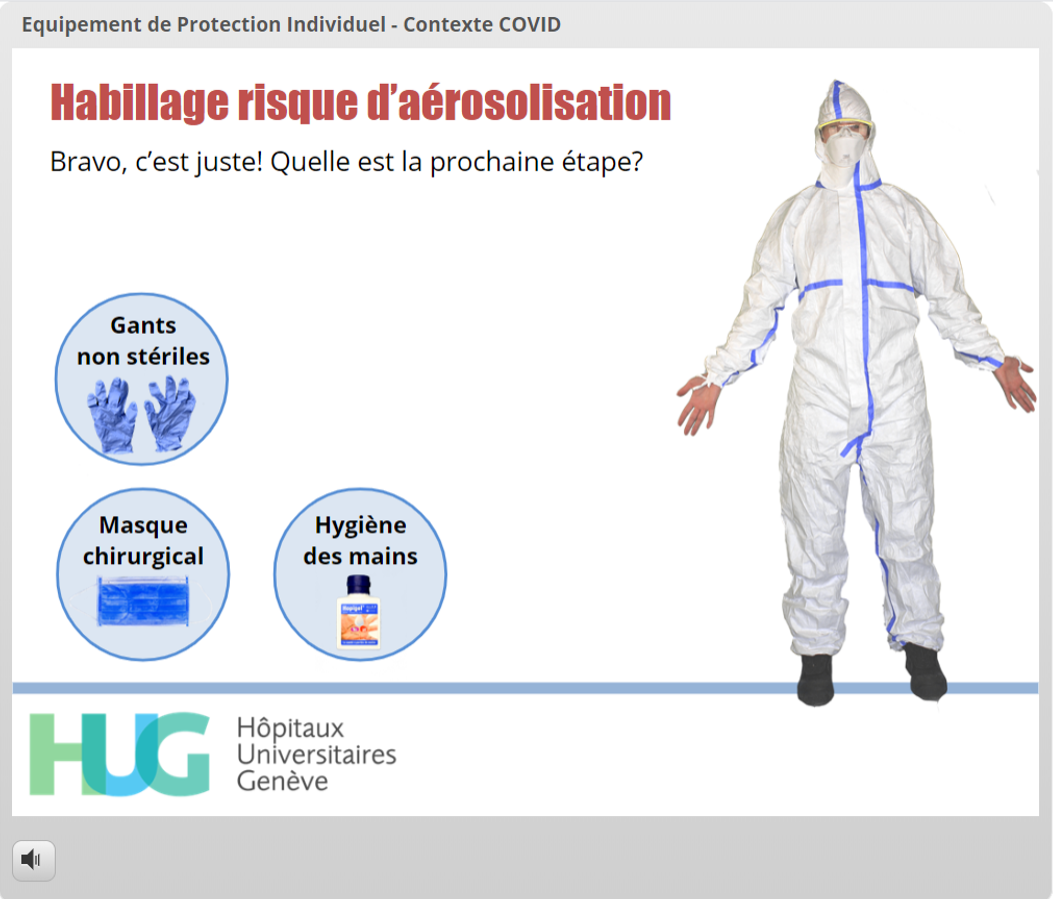
Change in the appearance of the prehospital provider to acknowledge the learner’s correct choice of PPE.

**Figure 14:**
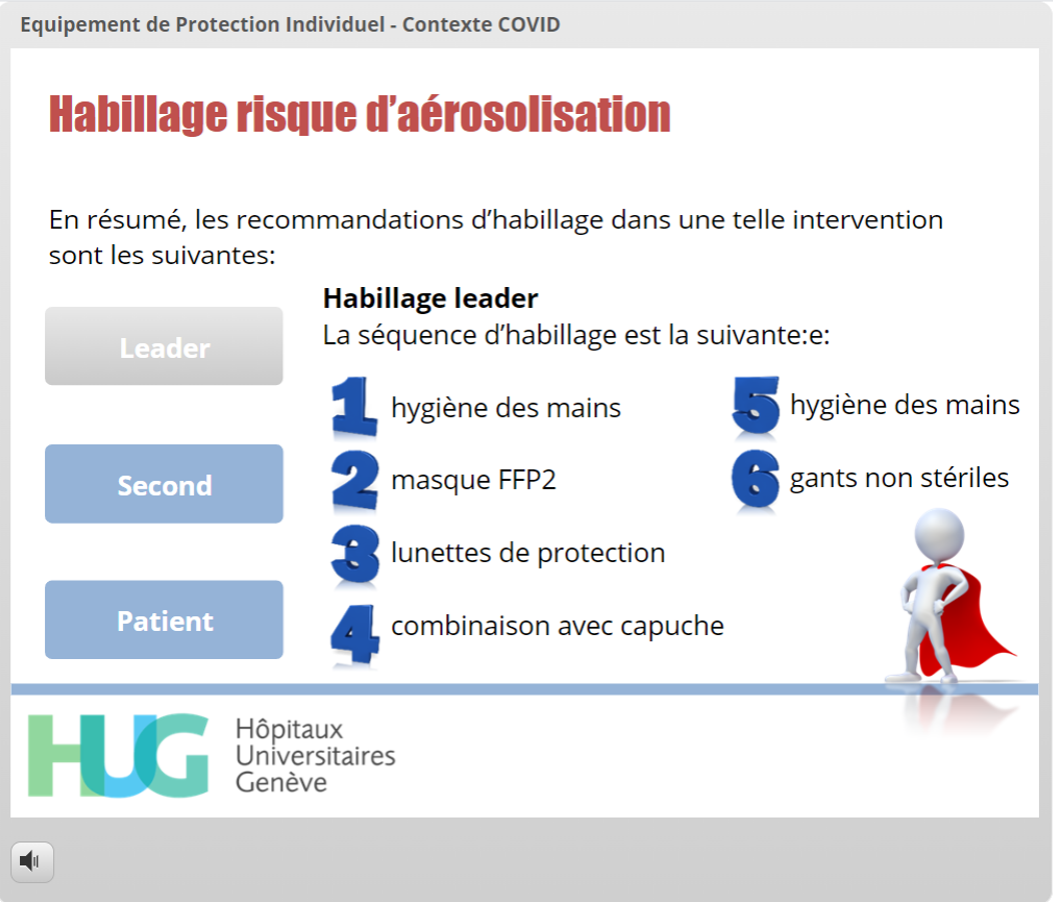
Repetition of the correct donning sequence for the lead prehospital provider in the context of a suspected or confirmed COVID-19 case with need for high-risk procedures.

**Figure 15:**
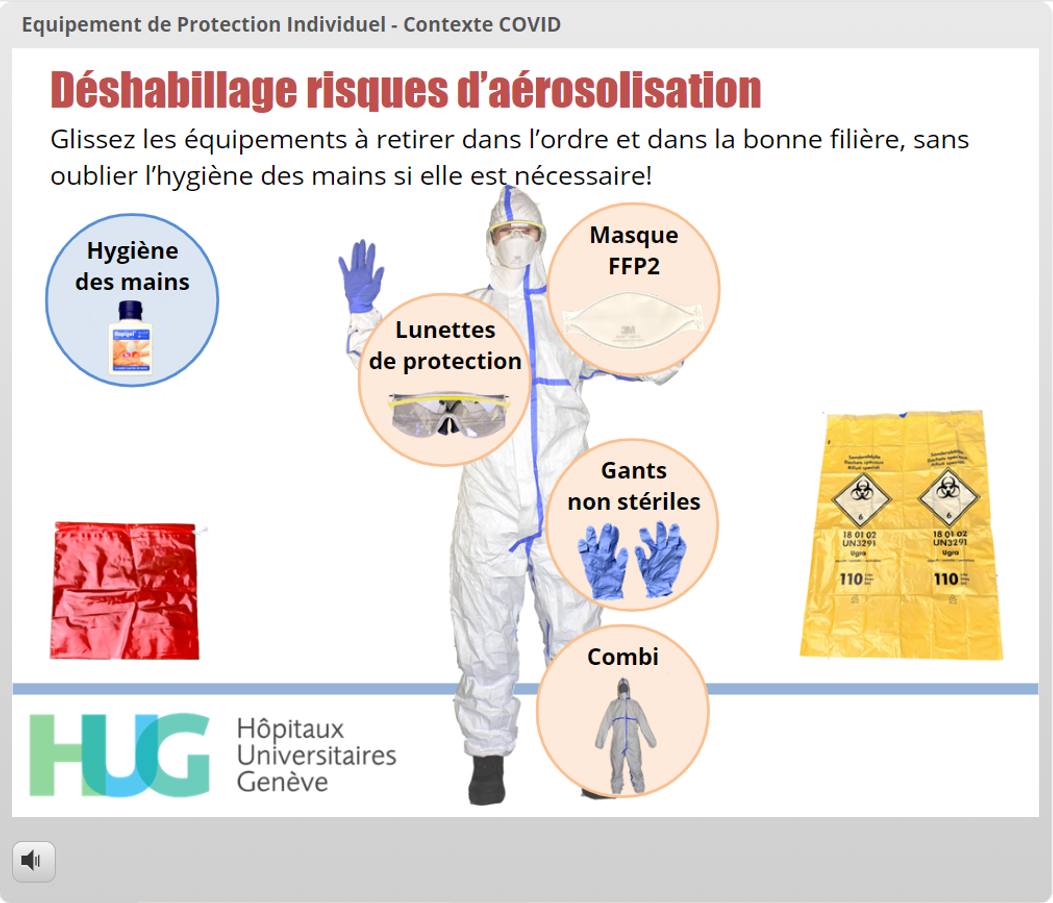
Gamification of the PPE doffing sequence. PPE worn by the prehospital provider must be dragged either to a biohazard trash bag (bottom right) or to a bag designed to hold reusable materials, such as protective goggles (bottom left).

**Figure 16:**
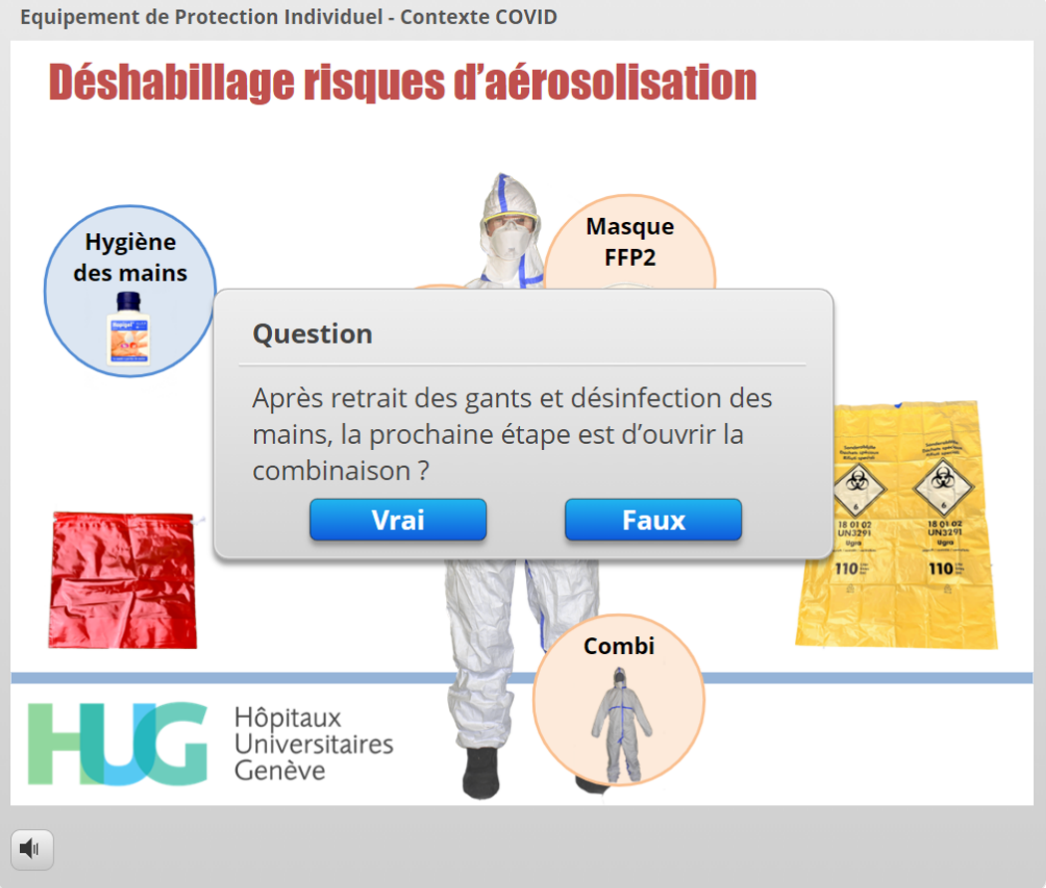
Use of a true/false interaction to assess the learner’s knowledge during particular steps in the doffing sequence.

When the choices were related to complex sequences, videos were launched automatically in lightbox slides (Figure 12). Once the correct PPE was chosen, the appearance of the EMS provider immediately changed to acknowledge the correct answer, thereby giving direct and visual feedback to the learner (Figure 13). Donning sequences were completed by slides repeating the correct donning sequences for the lead EMS provider (Figure 14) and by displaying the adequate PPE the EMS teammate and the patient should wear. The doffing sequences followed the same design, but in reverse: at the start of the sequence, the EMS provider wore PPE according to the risk setting. The circles containing PPE options were next to the provider, sometimes overlapping the photograph (Figure 15), and the learner was required to drag them away both in the right sequence and into the correct disposal bag (either a regular bin or a bag that could be stored to allow equipment decontamination and reuse). Specific PPE-related questions were asked using true/false interactions (Figure 16).

#### Module Validation

Once created, each sequence was thoroughly tested by all the other authors, who systematically provided feedback on each successive iteration. Usability issues were screened using the Davids heuristic evaluation procedure [27]. One major feature was added as a result of this procedure, namely the creation of a menu at the end of the module. This menu allowed learners to directly access any section of the module so they could review any specific element at will. Once all feedback was taken into account and the module was thoroughly debugged, a final version was validated by all the authors, among whom are chief ambulance and medical officers as well as infection prevention and control specialists who also act as consultants for the World Health Organization (WHO).

#### Module Availability

The gamified e-learning module can be accessed directly on the internet [23]. It can also be directly and freely downloaded from the website as a SCORM package or as a standalone web package, both of which are archived in independent ZIP files. The module can be reused freely under the Creative Commons 4.0 BY-NC-SA (Attribution-NonCommercial-ShareAlike) license [28].

## Discussion

### Principal Findings

A multiplatform gamified e-learning module designed using the SERES framework was created to help prehospital providers acquire knowledge regarding choice and use of PPE in the context of the COVID-19 pandemic [14]. The application of this framework to the development of a serious game has previously been described [29], and a pilot randomized controlled trial has shown promising results [30]. Although this framework was initially designed to help develop serious games, this study shows that it can also be used to create gamified content within materials that do not qualify as full-fledged games, and Deterding [31] indeed considers that gamification does not mandatorily imply the creation of a game but rather refers to “the use of game design elements in non-game contexts.” The main purposes of using gamification techniques are to create more engaging material and to improve user experience to achieve a particular goal. In this instance, one of the main goals was to enhance acquisition of knowledge and skills regarding PPE donning and doffing sequences.

The theoretical bases on which this module was built were not only taken from medical literature. Indeed, data related to the way health care workers or health care students absorb learning content is still scarce, and the model used to create this gamified module was partly derived from other fields, such as electronic engineering [20] and computer science [21]. The medical literature nevertheless provided some useful insights, among which are the potential shortcomings associated with the lack of video demonstration, which were identified in a previous trial [9]. The use of short videos targeting specific learning goals has been shown to enhance knowledge acquisition and retention and has demonstrated the ability to transfer information in many different fields; therefore, cutscenes were included in the e-learning module [32].

As Bloom’s revised taxonomy was used to define the target thinking skills, specific game mechanics were chosen to fit the learning objectives [15]. Although game mechanics based on rewards and action points are often used in gamification as well as in serious games, there is little data to suggest that their use enhances knowledge acquisition or retention. As these game mechanics are linked to the evaluating thinking skill, which was not elected during the design process, and as their inclusion in a serious game is not mandatory, we chose not to include them. In a similar way, badges, which are often used to keep learners engaged if multiple modules must be completed or if the course spans long periods of time, were not considered, as this was a single module that learners would be able to complete in a relatively short amount of time [33,34]. Moreover, rewards such as badges or action points may not yield the desired result, as learners may engage in the learning to obtain these rewards rather than to acquire specific skills or knowledge [35].

Integration of infection prevention experts early in the design of the module greatly helped us to gather the necessary media and ensure that the correct guidelines would be used during development. Validation of the content by these specialists, who also act as consultants for WHO, ensured the soundness of the elements taught in the module.

### Limitations

Despite the use of a solid scientific rationale and of the SERES framework during the entire development process, the main limitation of this paper is that this module has not yet been tested and its contribution as a learning tool cannot currently be assessed. A protocol for a randomized controlled trial has therefore been submitted to our regional ethics committee (Req-2020-00374). As this project does not involve patient data or participation, this committee has already issued a “declaration of no objection,” as such trials are not within the scope of Swiss federal law on human research [36].

Given the context of the COVID-19 pandemic and the need to quickly provide this gamified e-learning module to prehospital personnel, some of its features remain to be perfected. Indeed, more game design elements could be included to increase gamefulness. Key and selected elements from the RECIPE (Reflection-Engagement-Choice-Information-Play-Engagement) mnemonic for meaningful gamification developed by Scott Nicholson [35] could also serve as a building ground for development of further modules or even of a more comprehensive PPE course. However, some of these elements may not be relevant to this type of module, as they may imply too high a degree of freedom. Indeed, due to the need to achieve specific learning objectives, it is almost impossible to allow the learner to freely choose whether to engage in a specific and potentially crucial learning activity.

Other limitations must also be acknowledged. Indeed, although we strived to adhere to the most recent infection control guidelines, PPE type and use probably differs between EMS, and the contents of the module may not be compatible with some systems. Moreover, although we sought to enhance knowledge acquisition of PPE donning and doffing sequences through the use of game mechanics, these procedures may prove too complex to acquire without directly manipulating the PPE elements. This gamified e-learning module may therefore be better suited for a flipped classroom design [37,38]. However, the current need for social distancing prevents the organization of live training sessions, and this module may therefore be an acceptable surrogate until such training can be resumed.

Another limitation is that the module is currently only available in French. However, as this material is available under a Creative Commons license, it can now be used by many different institutions, ambulance companies, and hospitals. This may not only help spread knowledge regarding use of PPE but may also facilitate further research in the field. Last but not least, opportunities of distance teaching using gamified content may prove even more valuable than before, as social distancing may continue for many more months.

### Conclusion

A gamified e-learning module designed to promote knowledge and understanding of PPE use among prehospital health care workers during the COVID-19 pandemic was developed by following the SERES framework. The impact of this module should now be assessed by a randomized controlled trial.

## Abbreviations

COVID-19: coronavirus disease
EMS: emergency medical services
LM-GM: learning mechanics-game mechanics
MeSH: medical subject headings
PPE: personal protective equipment
SARS-CoV-2: severe acute respiratory syndrome coronavirus 2
WHO: World Health Organization
